# Prevalence of Financial Considerations Documented in Primary Care Encounters as Identified by Natural Language Processing Methods

**DOI:** 10.1001/jamanetworkopen.2019.10399

**Published:** 2019-08-30

**Authors:** Meliha Skaljic, Ihsaan H. Patel, Amelia M. Pellegrini, Victor M. Castro, Roy H. Perlis, Deborah D. Gordon

**Affiliations:** 1 Perelman School of Medicine, University of Pennsylvania, Philadelphia; 2 Mossavar-Rahmani Center for Business and Government, Harvard Kennedy School, Cambridge, Massachusetts; 3 Center for Quantitative Health, Division of Clinical Research, Massachusetts General Hospital, Harvard Medical School, Boston; 4 Center for Genomic Medicine, Massachusetts General Hospital, Harvard Medical School, Boston

## Abstract

**Importance:**

Quantifying patient-physician cost conversations is challenging but important as out-of-pocket spending by US patients increases and patients are increasingly interested in discussing costs with their physicians.

**Objective:**

To characterize the prevalence of financial considerations documented in narrative clinical records of primary care encounters and their association with patient-level features.

**Design, Setting, and Participants:**

This cohort study applied natural language processing to narrative clinical notes obtained from electronic health records for adult primary care visits. Participants included patients aged 18 years and older with at least 1 primary care visit for an annual preventive examination at outpatient clinics at a US academic health system between January 2, 2008, and July 30, 2013. Data were analyzed in March 2019.

**Main Outcomes and Measures:**

Presence of financial content documented in narrative clinical notes.

**Results:**

The data set included 222 457 primary care visits for 46 244 individuals aged 18 years and older; 30 556 patients (60.1%) were female, 27 869 patients (60.3%) were white, and the mean (SD) age was 51.3 (17.7) years. In total, 6058 patients (13.1%) had at least 1 narrative clinical note indicating a financial conversation with their physician. In fully adjusted regression models, the odds of having a financial note were greater among patients with Medicare (odds ratio [OR], 1.27; 95% CI, 1.15-1.41; *P* < .001) or Medicaid (OR, 1.43; 95% CI, 1.25-1.64; *P* < .001) insurance, those residing in zip codes with lower median income (OR, 0.97; 95% CI, 0.96-0.98; *P* < .001), black individuals (OR, 1.40; 95% CI, 1.28-1.53; *P* < .001), Hispanic individuals (OR, 1.10; 95% CI, 1.01-1.20; *P* = .03), and those who were unmarried (OR, 1.23; 95% CI, 1.15-1.33; *P* < .001).

**Conclusions and Relevance:**

Cost considerations were more likely to be noted in annual preventive examinations than previously observed in intensive care unit admissions, but still infrequently. Associations with particular patient subgroups may indicate differential financial burden or willingness to discuss financial concerns.

## Key Points

**Question:**

How often are cost considerations documented in narrative clinical notes in a primary care setting?

**Findings:**

In this cohort study of a data set including 222 457 outpatient primary care notes for 46 244 patients at a large academic medical center, 13.1% of patients had at least 1 note indicating a financial conversation with their physician. Specific socioeconomic features were associated with the presence of documented cost considerations.

**Meaning:**

Although the literature suggests that patients desire to discuss health costs with their physicians, these conversations remain infrequent.

## Teaser

This cohort study applies natural language processing to narrative clinical notes obtained from electronic health records of a US academic health system to analyze the frequency of financial conversations during adult primary care visits.

## Introduction

Household out-of-pocket spending on health care has been increasing steadily over recent years in the United States, driven at least in part by the growing popularity of high-deductible health plans.^1^ In response, physicians are becoming increasingly mindful of patient spending: some physician groups explicitly advocate for the consideration of costs in their clinical guideline documents,^2^ and a survey^3^ revealed that 84% of oncologists incorporate patient out-of-pocket costs into treatment recommendations. There is also a growing body of literature examining the prevalence and content of cost-related conversations between patients and physicians. Most US individuals, up to 70% according to a large national study from 2017,^4^ are interested in discussing cost with their physician, although current estimates of the actual prevalence of cost conversations vary widely, from 4% to 65%.^5^ These previous estimates largely relied on either survey data, which may be biased, or recorded clinic interactions, which may result in the disruption of care.

As an alternative to these existing methods, in our previous work,^6^ we applied machine learning to electronic health records to develop a highly discriminative model identifying the presence and nature of financial considerations in intensive care unit (ICU) clinical notes. Here, we sought to understand the prevalence of such conversations in a cohort likely to be more representative of medicine as a whole, particularly outpatient medicine. We applied the model trained on the ICU data to outpatient primary care notes from a large academic medical center. As in the ICU setting, we also aimed to understand the extent to which these conversations might be associated with patient-level sociodemographic features.

## Methods

### Cohort Generation and Study Design

We queried the Partners Research Patient Data Registry to identify all adult primary care visits for annual preventive examinations at Massachusetts General Hospital and Brigham and Women’s Hospital between January 2, 2008, and July 30, 2013 (data beyond 2013 were not available because of migration to a new electronic medical record system). To control for the type of visit, we sought to capture routine primary care visits rather than problem-based visits: adult annual preventive examinations were identified using *Current Procedural Terminology, Fourth Edition* codes 99204, 99205, 99214, and 99215. We identified 222 457 primary care visits for 46 244 individuals aged 18 years and older. In addition to narrative notes, sociodemographic features were extracted from structured data, including age, sex, marital status, race/ethnicity, insurance type, and median household income based on 2013 census data^7^ imputed from zip code, generating a data mart using i2b2 server software.^8^

The Partners HealthCare Human Research Committee approved the study protocol and waived the requirement for informed consent under 45 CFR 46.116 because no participant contact was required in this study based on secondary use of data arising from routine clinical care. This study follows the Strengthening the Reporting of Observational Studies in Epidemiology (STROBE) reporting guideline.

### Model Training

We have previously described the derivation of the natural language processing (NLP) model used to classify notes.^6^ In brief, the notes labeled for the presence of financial conversations from the previous study were used to train a random forest classifier with Python’s scikit-learn package (sklearn.ensemble.RandomForestClassifier, version 0.20.0).^9^ These notes were randomly split into a training set of 5021 of 5579 notes (90.0%) for model development and tuning, and a testing set of 558 (10.0%) was used to confirm the model’s ability to generalize. The number of notes used to train the model was slightly higher than that in the previous study because nonindex admission notes were also included to allow for more data to train on. As previously discussed,^6^ standard NLP techniques, including removing punctuation and English-language stop words, stemming, generating unigrams and bigrams, creating a term frequency–inverse document frequency matrix from these unigrams and bigrams, and using principal components analysis to reduce the dimensionality of this matrix, were used to transform the text before model training.

Areas under the curve calculated by using the trapezoidal rule, precision, and recall were used as the primary evaluation metrics for discrimination on the training and test sets. On the independent testing set, the area under the curve exceeded 0.96. Precision and recall were calculated at different thresholds of the model’s output probability to determine which threshold struck the best balance for our use case and would, therefore, be used on the outpatient data set. In this case, a threshold of 0.70 was chosen for subsequent analysis because it yielded both precision and recall values greater than 0.92 on the testing set.

### Model Evaluation on Outpatient Data Set

The 222 457 clinical notes in the outpatient data set were preprocessed in exactly the same way as the training set. To validate the performance of the model on the outpatient data set, 1 author manually tagged 100 of these notes to determine a reference standard. On these 100 notes, the area under the curve was 0.83, and precision and recall were 0.83 and 0.93, respectively, at the predetermined 0.70 threshold. One author then manually tagged 300 patient notes identified by the model as having a documented financial conversation into further subcategories according to the nature of the conversation.

### Statistical Analysis

Summary statistics, including proportion for categorical features and mean (SD) for continuous features, were computed for each group (ie, patients who had engaged in financial conversations and patients who had not) using Python’s pandas (version 0.23.4)^10^ and scipy (version 1.1.0)^11^ packages. No data were missing. Comparisons used χ^2^ tests or single-sample, unpaired, 2-tailed *t* tests, as appropriate. After these univariate analyses, binomial logistic regression models using Python’s statsmodel (version 0.9.0),^12^ adjusted for the total number of notes associated with the patient, age, sex, race/ethnicity, insurance type, and zip code median income at earliest recorded visit, were fit with the presence or absence of financial discussion as the dependent variable to estimate the independent effect of each sociodemographic feature in terms of odds ratios (ORs) and 95% confidence intervals. All reported *P* values are 2-sided, with nominal significance considered to be uncorrected *P* < .05.

## Results

The data set as a whole included 222 457 outpatient notes for 46 244 patients seen between January 2, 2008, and July 30, 2013. Of these 46 244 patients, 30 556 (60.1%) were female, 27 869 (60.3%) were white, and the mean (SD) age was 51.3 (17.7) years. In total, 6058 patients (13.1%) were found to have at least 1 note over this period indicating a financial conversation with their physician. For each year between 2008 and 2013, 7.2%, 7.6%, 7.6%, 8.2%, and 6.7% of patients, respectively, had at least 1 cost-related note; each of these percentages is lower than the overall estimate because many patients had multiple years of notes.

Table 1 describes the sociodemographic features of the study population. In univariate comparisons, compared with those without financial conversations, patients with financial conversations documents in their notes were older (mean [SD] age, 54.1 [16.4] vs 49.9 [17.5] years; *t* = 19.9; *P* < .001) and resided in lower-income areas (mean [SD] zip code median income, $70 684 [$28 377] vs $76 782 [$30 366]; *t* = −16.6; *P* < .001) than patients without such notes. Financial notes were also more likely to appear in the records of women (4170 of 30 556 patients [13.7%] vs 1888 of 15 688 patients [12.0%]; χ^2^ = 23.5; *P* < .001), individuals of Hispanic (1223 of 7776 [15.7%] vs 4835 of 38 468 [12.6%]; χ^2^ = 56.4; *P* < .001) and black (975 of 5392 [18.1%] vs 5083 of 40 852 [12.4%]; χ^2^ = 132.6; *P* < .001) race/ethnicity, those who were single (1982 of 14 271 [13.9%] vs 4076 of 31 973 [12.8%]; χ^2^ = 11.2; *P* < .001), and those with Medicare (643 of 3529 [18.2%] vs 5415 of 42 715 [12.7%]; χ^2^ = 87.5; *P* < .001) and non-Medicare (1729 of 8861 [19.5%] vs 4239 of 37 383 [11.6%]; χ^2^ = 395.2; *P* < .001) government insurance.

**Table 1.** Sociodemographic Features Associated With Presence or Absence of Financial Notes for 46 244 Patients

Feature	Financial Note (n = 6058)		No Financial Note (n = 40 186)		Test Statistic^a^	*P* Value
	Participants, No. (%)	Reference, No. (%)	Participants, No. (%)	Reference, No. (%)		
Age, mean (SD), y	54.1 (16.4)		49.9 (17.5)		19.9	<.001
Zip code median income, mean (SD), $10 000	7.07 (2.8)		7.68 (3.0)		−16.6	<.001
Female	4170 (13.7)	1888 (12.0)	26 386 (86.4)	13 800 (88.0)	23.5	<.001
Race/ethnicity						
White	3227 (11.6)	2831 (15.4)	24 642 (88.4)	15 544 (84.6)	142.2	<.001
Hispanic	1223 (15.7)	4835 (12.6)	6553 (84.3)	33 633 (87.4)	56.4	<.001
Black	975 (18.1)	5083 (12.4)	4417 (81.9)	35 769 (87.6)	132.6	<.001
Other	511 (13.8)	5547 (13.0)	3192 (86.2)	36 994 (87.0)	1.7	.20
Asian	122 (8.1)	5936 (13.3)	1382 (91.9)	38 804 (86.7)	33.5	<.001
Marital status						
Married	2654 (11.0)	3404 (15.4)	21 508 (89.0)	18 678 (84.6)	198.6	<.001
Single	1982 (13.9)	4076 (12.8)	12 289 (86.1)	27 897 (87.3)	11.2	<.001
Divorced	570 (19.0)	5488 (12.7)	2435 (81.0)	37 751 (87.3)	96.7	<.001
Widowed	526 (19.1)	5532 (12.7)	2231 (80.9)	37 955 (87.3)	91.5	<.001
Other or unknown	326 (15.9)	5732 (13.0)	1723 (84.1)	38 463 (87.0)	14.6	<.001
Insurance type						
Private	2716 (10.5)	3342 (16.4)	23 170 (89.5)	17 016 (83.6)	350.8	<.001
Public, other	1729 (19.5)	4329 (11.6)	7132 (80.5)	33 054 (88.4)	395.2	<.001
Other or unknown	710 (11.8)	5348 (13.3)	5323 (88.2)	34 863 (86.7)	10.7	.001
Public, Medicare	643 (18.2)	5415 (12.7)	2886 (81.8)	37 300 (87.3)	87.5	<.001
Public, Medicaid	260 (13.4)	5798 (13.1)	1675 (86.6)	38 511 (86.9)	0.2	.68

^a^ Statistics are *t* value for continuous variables and χ^2^ for categorical variables.

Multivariable logistic regression was used to determine the independent association of sociodemographic factors with the likelihood of a financial note (Table 2). There continued to be significant associations with neighborhood income (lower zip code median income, OR, 0.97; 95% CI, 0.96-0.98; *P* < .001), nonwhite race/ethnicity (Hispanic individuals, OR, 1.10; 95% CI, 1.01-1.20; *P* = .03; black individuals, OR, 1.40; 95% CI, 1.28-1.53; *P* < .001), marital status (unmarried, OR, 1.23; 95% CI, 1.15-1.33; *P* < .001), and insurance type (Medicare, OR, 1.27; 95% CI, 1.15-1.41; *P* < .001; Medicaid, OR, 1.43; 95% CI, 1.25-1.64; *P* < .001). However, age (OR, 1.00; 95% CI, 1.00-1.00; *P* = .05) and female sex (OR, 1.05; 95% CI, 0.99-1.12; *P* = .10) were no longer statistically significantly associated with the presence of financial notes.

**Table 2.** Association Between Sociodemographic and Clinical Features and Presence of Financial Notes for 46 244 Patients

Feature	OR (95% CI)		*P* Value
	Unadjusted	Adjusted^a^	
Note count, No.	1.13 (1.12-1.13)	1.12 (1.12-1.13)	<.001
Age, y	1.02 (1.01-1.02)	1.00 (1.00-1.00)	.05
Zip code median income, $10 000	0.93 (0.92-0.94)	0.97 (0.96-0.98)	<.001
Female	1.15 (1.09-1.22)	1.05 (0.99-1.12)	.10
Race			
White	1 [Reference]	1 [Reference]	
Hispanic	1.29 (1.21-1.39)	1.10 (1.01-1.20)	.03
Black	1.56 (1.44-1.68)	1.40 (1.28-1.53)	<.001
Other	1.10 (1.00-1.22)	1.11 (0.99-1.24)	.07
Asian	0.58 (0.48-0.70)	0.85 (0.70-1.03)	.10
Marital status			
Married	1 [Reference]	1 [Reference]	
Single	1.10 (1.04-1.17)	1.23 (1.15-1.33)	<.001
Divorced	1.60 (1.46-1.77)	1.48 (1.33-1.65)	<.001
Widowed	1.29 (1.14-1.46)	1.04 (0.92-1.17)	.57
Other or unknown	1.61 (1.45-1.77)	1.47 (1.29-1.68)	<.001
Insurance type			
Private	1 [Reference]	1 [Reference]	
Public, other	1.81 (1.71-1.93)	1.47 (1.36-1.59)	<.001
Other or unknown	0.88 (0.81-0.97)	1.25 (1.13-1.38)	<.001
Public, Medicare	1.70 (1.57-1.85)	1.27 (1.15-1.41)	<.001
Public, Medicaid	1.23 (1.09-1.39)	1.43 (1.25-1.64)	<.001
Intercept	0.06 (0.05-0.07)	0.06 (0.05-0.07)	<.001

Abbreviation: OR, odds ratio.

^a^ Adjusted for the total number of notes associated with the patient, age, sex, race/ethnicity, insurance type, and zip code median income at earliest recorded visit.

Finally, 300 of the notes identified by the NLP model as having documented financial conversations were then further subcategorized by the nature of the discussion (Table 3); 271 (90.3%) were true discussions of financial matters. Of these notes, 191 (70.5%) included a discussion of health insurance, 132 (48.7%) and 78 (28.8%) documented a change in treatment plan or medication, respectively, and 47 (17.3%) mentioned non–health-related financial concerns (eg, unemployment, legal troubles, and child care expenses).

**Table 3.** Nature of Documented Financial Conversations of 300 Representative Notes

Type of Note	Notes, No. (%)
Tagged notes (n = 300)	
True financial conversation	271 (90.3)
Nonfinancial conversation	29 (9.7)
Financial conversations (n = 271)^a^	
Addressed health insurance	191 (70.5)
Treatment changed as a result	132 (48.7)
Medication changed as a result	78 (28.8)
Non–health-related financial challenges	47 (17.3)

^a^ Numbers in the categories do not total 271 because conversations could be assigned to more than 1 category.

## Discussion

In application of a previously validated NLP model to a data set of 222 457 outpatient primary care notes, we found that 13.1% of 46 244 patients had at least 1 documented conversation with their physician that referenced financial considerations. The percentage of patients with at least 1 cost-related conversation in a given year was fairly constant at 6.7% to 8.2% per year. In adjusted analyses, these discussions were more likely for unmarried individuals, individuals residing in areas with lower median household incomes, individuals with government health insurance, and individuals of black and Hispanic race/ethnicity.

Estimates of the prevalence of financial conversations vary widely across the literature; for a summary, see the article by Hunter et al.^5^ These estimates include 30% of dialogue transcripts from specialist outpatient visits with a mention of cost,^1^ 15% of primary care physicians in California reporting discussing costs with their patients most or all of the time,^13^ and 40% of African American women from a single center reporting having a conversation about the cost of their asthma care with their physician.^14^ In our own previous work^6^ applying NLP to ICU clinical notes, we reported that 4.2% of patients had at least 1 note reflecting financial considerations during their stay. Clearly, there are many reasons why the reported estimates may differ, including the method of data collection, nature of the clinical context, and criteria used to define a financial conversation.

Of note, all prior work on this subject is based on either survey data or recorded dialogue from clinic visits. To our knowledge, this study is the first to use NLP to arrive at an estimate of the prevalence of financial conversations in the outpatient setting. That our estimate falls within the range of previously published work not only validates the accuracy of this method but also illustrates how machine learning can be applied to large data sets to answer questions more efficiently and cost-effectively than ever before. Furthermore, we demonstrate how an existing model, in this case an NLP model trained on the ICU notes from our previous work,^6^ can be repurposed for a second task. This strategy, known as transfer learning, can overcome issues in which a clinical data set is too large to be relabeled entirely (or too small for a model to be trained on it effectively).^15^ The strong performance of the ICU model on the outpatient visit notes suggests that this model can be successfully applied to other data sets in the future.

In a random sample of notes with documented financial conversations, we found that 48.7% of conversations resulted in a change to treatment and 28.8% resulted in a change of medication. The finding that patient-physician discussions of cost can result in a change of management is in line with other research suggesting that physicians are increasingly incorporating patient out-of-pocket spending into their decisions-making.^2,3,16^ Whether these changes are associated with health outcomes remains to be determined.

Unsurprisingly, our results demonstrate that socioeconomic factors are associated with the presence of cost conversations. Single patients, individuals residing in zip codes with lower median incomes, and those with government insurance including Medicaid likely experience a greater economic burden associated with medical care compared with other groups and, thus, are more likely to discuss cost with their physician. These findings may inform physician behavior and training; for example, medical students and residents may be taught which medications are more or less likely to be approved by public insurance plans. However, the association between Medicare insurance and the presence of financial notes is more surprising, because Medicare is intended to protect senior citizens against medical expenditure risk. We postulate that this may be reflective of global survey findings^17^ showing that US seniors face more financial barriers to care, despite nearly universal access via Medicare, than their peers in other high-income countries. It may also reflect a decline in financial literacy among seniors, which may increase economic anxiety in this group.^18^

We also found that black and Hispanic individuals are more likely to discuss cost with their physicians, as evident in both crude and adjusted models. A recent large patient survey^19^ found similar results: black and Hispanic individuals were more likely to be aware of price before receiving care, to investigate out-of-pocket spending, and to compare costs across physicians. This result may be confounded by other variables that drive either increased financial burden of care or increased desire to discuss financial concerns for black and Hispanic individuals. On the other hand, we also recognize that if these conversations were primarily initiated by the physician and not the patient, they may reflect unconscious bias by physicians that black and Hispanic individuals have greater economic burden compared with others.^20^ Drawing a meaningful conclusion from this result is predicated on understanding in greater detail the types of conversations that occur and which party initiated them.

Our findings add to the existing research exploring the role of financial considerations in encounters between patients and physicians. Continued study of the nature of these conversations can guide future efforts to improve communication between patients and physicians, particularly as a new generation of physicians is being trained.

### Limitations

We note multiple limitations in this work. Our model was initially trained on inpatient ICU notes, which may involve language and themes that are different from those of outpatient settings. It is also likely that we underestimate the true prevalence of patient-physician financial conversations because some discussions may not be documented in clinical notes or may take place with front desk or billing staff instead of the physician. The present data also do not allow investigation of physician-level features that likely are associated with the probability of such discussions. In addition, other unmeasured variables (eg, medical comorbidities and the presence or absence of an acute problem at time of visit) are likely to be associated with the probability of financial discussion. Also, our analysis was limited to a single academic medical center; future applications of the model to data from other sites may reveal interesting regional and institutional differences in the presence of cost conversations.

## Conclusions

This study shows the feasibility of transferring a previously developed NLP model to identify documented financial conversations in narrative clinical notes and provides evidence to suggests that these conversations can influence medical decision-making by physicians. The likelihood of documented financial conversations is associated with sociodemographic factors, including marital status, insurance type, and race/ethnicity. This approach adds to the range of strategies for investigating the extent and nature of financial conversations, as a means of better characterizing the financial burden of health care for individual patients.
